# Immigrants in the one percent: The national origin of top wealth owners

**DOI:** 10.1371/journal.pone.0172876

**Published:** 2017-02-23

**Authors:** Lisa A. Keister, Brian Aronson

**Affiliations:** Duke University, Department of Sociology, Durham, North Carolina, United States of America; Iowa State University, UNITED STATES

## Abstract

**Background:**

Economic inequality in the United States is extreme, but little is known about the national origin of affluent households. Households in the top one percent by total wealth own vastly disproportionate quantities of household assets and have correspondingly high levels of economic, social, and political influence. The overrepresentation of white natives (i.e., those born in the U.S.) among high-wealth households is well-documented, but changing migration dynamics suggest that a growing portion of top households may be immigrants.

**Methods:**

Because no single survey dataset contains top wealth holders and data about country of origin, this paper uses two publicly-available data sets: the Survey of Consumer Finances (SCF) and the Survey of Income and Program Participation (SIPP). Multiple imputation is used to impute country of birth from the SIPP into the SCF. Descriptive statistics are used to demonstrate reliability of the method, to estimate the prevalence of immigrants among top wealth holders, and to document patterns of asset ownership among affluent immigrants.

**Results:**

Significant numbers of top wealth holders who are usually classified as white natives may be immigrants. Many top wealth holders appear to be European and Canadian immigrants, and increasing numbers of top wealth holders are likely from Asia and Latin America as well. Results suggest that of those in the top one percent of wealth holders, approximately 3% are European and Canadian immigrants, .5% are from Mexico or Cuban, and 1.7% are from Asia (especially Hong Kong, Taiwan, Mainland China, and India). Ownership of key assets varies considerably across affluent immigrant groups.

**Conclusion:**

Although the percentage of top wealth holders who are immigrants is relatively small, these percentages represent large numbers of households with considerable resources and corresponding social and political influence. Evidence that the propensity to allocate wealth to real and financial assets varies across immigrant groups suggests that wealth ownership is more global than previous research suggests and that immigrant groups are likely to become more prevalent in top wealth positions in the U.S. As the representation of immigrants in top wealth positions grows, their economic, social, and political influence is likely to increase as well.

## Introduction

Increasing economic inequality in the United States has drawn considerable attention to the small number of extremely affluent households who control most financial resources [1–4]. These households–the one percent–have most commonly been defined only by their incomes [1, 4–6], but it is now clear that wealth (net worth, or total assets less total debts) is even more unequally distributed than income [7–9]. The top one percent receives about 20% of total household income, but the top one percent by wealth owns 35% of net worth and 38% of financial assets. Consistent with this, the Gini coefficient for income is approximately .50, but the wealth Gini is more than .80. The gap between top wealth holders and the median household underscores this concentration: it takes nearly $8 million in net worth and $4 million in financial assets to be in the top one percent, but the median household owns just over $80,000 in net worth and $17,000 in financial assets (authors’ estimates from the Survey of Consumer Finances).

Although trends in inequality are now clear, scholars know little about the small number of households at the top of the wealth distribution. One particularly important gap is the lack of information about the national origin of top wealth holders. The overrepresentation of white natives (i.e., born in the U.S.) among high-wealth households is well-documented [10–13], but the ethnic and national origin of nearly 10% of top wealth holders is ambiguous [2]. Immigration dynamics suggest that many of these unspecified households may, indeed, be foreign-born. Thirteen percent of the United States’ population (40 million people) is foreign-born [14], and the foreign-born population has changed dramatically in recent decades from an older, mostly European-born population to a younger, Latin American and Asian population [15]. Moreover, some immigrant groups are highly-selected on traits such as education that lead to wealth accumulation; for example, large influxes of Mainland Chinese and Indian immigrants with relatively high education levels may have increased the representation of these groups at the top of the wealth distribution [15–17]. Other foreign-born groups enter the U.S. with little education, income, or occupational experience but are upwardly mobile over time on many predictors of wealth ownership [18–20].

This paper provides the first detailed estimates of the national origin of top wealth holders in the U.S. and explores how asset ownership varies across affluent immigrant groups. It is important to understand who occupies top wealth positions because these households have important economic, social, and political advantages that are potentially more far-reaching than those associated with income. Moreover, because wealth can create more wealth and can be passed to future generations, even small numbers of households with the potential to accumulate considerable wealth can portend long-term changes in the wealth distribution.

Because no single survey dataset contains top wealth holders and data about country of origin, this paper uses two publicly-available data sets: the Survey of Consumer Finances (SCF) and the Survey of Income and Program Participation (SIPP). Multiple imputation is used to impute country of birth from the SIPP into the SCF. Descriptive statistics are used to demonstrate reliability of the method, to estimate the prevalence of immigrants among top wealth holders, and to document patterns of asset ownership among affluent immigrants.

Results show that significant numbers of top wealth holders who are usually classified as white natives may be immigrants. Many top wealth holders appear to be European and Canadian immigrants, but increasing numbers of top wealth holders may be from Asia and Latin America as well. Of those in the top one percent of wealth holders, approximately 3% are European and Canadian immigrants, .5% are from Mexico or Cuban, and 1.7% are from Asia (especially Hong Kong, Taiwan, Mainland China, and India). Ownership of key assets varies considerably across affluent immigrant groups.

Although the percentage of top wealth holders who are immigrants is relatively small, these percentages represent large numbers of households with considerable resources and corresponding influence. Evidence that the propensity to allocate wealth to real and financial assets varies across immigrant groups suggests that wealth ownership is more global than previous research suggests and that immigrant groups are likely to become more prevalent in top wealth positions in the U.S.

## Background

Wealth is among the most significant and consequential indicators of household well-being, and for immigrants, wealth is also an important indicator of socioeconomic assimilation. Saved assets can ensure a secure retirement, mitigate the effects of unemployment and other income shocks, provide a safe living environment, and create educational and occupational opportunities. At higher levels, wealth can also provide significant political and social influence [21]. Wealth has important long-term effects because assets can create more assets when the interest and dividends they earns are reinvested, and wealth can be passed to future generations to extend its benefits indefinitely. For immigrants, many of the standard indicators of incorporation (e.g., education, income, family processes, language, and legal status) are reflected in wealth. Home and business ownership often hold particular significance for immigrants and reflect many immigrants’ conceptions of mobility and becoming American [22–24].

Understanding the top of the wealth distribution is particularly important because asset ownership is highly skewed. Researchers have begun to coalesce around studying the top one percent of wealth owners, a cutoff that provides a metric for research on advantaged households that is somewhat comparable to the poverty line used in research on the disadvantaged. Using other cutoffs to study top groups—such as the top five or ten percent—is also useful, but the degree to which wealth is concentrated at the top of the distribution suggests that studying those in the top one percent offers the greatest insight into those with the most control of this resource. Studying the one percent has its origins in early work on income and savings [25], but it did not become common in academic research until the early 2000s [26, 27]. The term entered has become increasingly relevant in popular discourse following the Occupy Wall Street movement which renewed public interest in inequality and the degree to which both wealth and income are concentrated [28–30]. Understanding whether immigrants are represented among top wealth holders is consequential because the wealthy have significant social and political influence and changes in the composition of those in top wealth positions reflects changes in the distribution of power and influence. Moreover, the wealthy tend to pass their wealth to future generations which can lead to long-term changes in immigrant social and economic status.

Given that the immigrant population of the U.S. is large and that immigrants are over-selected for attributes that encourage wealth accumulation, it is likely that significant number of top wealth holders are foreign-born. For instance, saving motives and behaviors differ for immigrants compared to native-born ethnic groups in ways that the top of the wealth distribution includes substantial numbers of immigrants. Although natives tend to save more than immigrants (particularly precautionary saving in anticipation of income shocks), immigrants tend to be hyperselected for traits that encourage saving [31–33]. There is also evidence that some of the difference between natives and immigrants in saving behavior may reflect immigrant remittances to the home country which immigrants may use as a saving vehicle and to reduce risk [31, 34]. Remitting tends to be highest for new immigrants; however, as immigrants become more embedded in social networks in the U.S., remittances tend to decline [35–38]. Thus, immigrants who have been in the U.S. for some time are likely to have both the traits and the resources necessary for significant wealth accumulation. Relative to natives, many immigrants also have greater incentives to save for particular purposes including their children’s college educations [39], homeownership [18, 22], and business startup [40, 41].

Evidence regarding migration patterns to the U.S. from particular countries also suggests that certain groups are likely to be represented among top wealth owners. For example, current estimates indicate that the vast majority of top wealth holders are white (about 92%), and such estimates are usually interpreted to mean native-born whites [2, 42]. However, at least some of these households are likely to be Caucasian (white) immigrants. Although their numbers have been decreasing, white immigrants from Canada and various European countries (including Russia) have been among the largest groups of immigrants to the U.S. for decades. Importantly, immigrants from these regions, on average, have education levels, incomes, professional skills, experience in high-status occupations, and entrepreneurial ambitions that are high compared to both the populations in their countries of origin and the U.S. population [43–45]. Given that these traits predict wealth ownership, the odds of entry into top wealth positions are likely high for white Canadian and European immigrants. Since Canadian and European immigrants have a relatively high propensity to hold assets in the home country and to transfer assets accumulated in the U.S. back to the home country, the presence of people from these regions in top wealth positions also has implications for both the current and future distribution of household wealth globally.

Perhaps more importantly, changing immigration dynamics suggest that many of the foreign-born top wealth holders are likely to be Latino and Asian immigrants [14, 46, 47]. Whereas previous generations of immigrants largely arrived from Europe and Canada, most immigrants to the U.S. since the 1970s are Latin American and Asian, in part, reflecting changes to U.S. immigration law [14, 46]. Amendments to the Immigration Act in 1965 eliminated national origin quotas and, as a result, encouraged immigration from regions such as Latin America and Asia from which immigration was previously less common. The new law also created restrictions on immigration by hemisphere, although these hemisphere quotas were later abolished in favor of overall quotas on immigrant numbers. The 1965 law also relaxed restrictions on certain categories of immigrants such as those with family connections or with special skills needed by U.S. industries. Further changes to the makeup of the foreign-born population resulted from the 1986 Immigration Reform and Control Act (IRCA) that gave legal status to millions of undocumented immigrants and increased the cap on immigration. These changes precipitated dramatic changes in the demographics of those entering the U.S. as immigrants, and many of the most significant changes were not apparent until a couple decades following the initial legislative updates.

Among the most notable changes to immigration that resulted from legal reform occurred among the Latino population. Latinos currently make up about 1.2% of top wealth owners in the U.S. [2], and it is likely that a sizable portion of these households are Mexican American. Two-thirds of American Latinos identify as Mexican [47], and Mexican Americans have been at the heart of controversies about whether immigrants can and do assimilate [48, 49]. Although Mexicans tend to be disadvantaged and to have higher rates of undocumented status even among immigrants [22, 43, 50], there is mounting evidence that many Mexican immigrants–particularly those who have spent sufficient time in the U.S.–have many of the traits that lead to wealth accumulation particularly after spending sufficient time in the U.S. [20, 22]. For example, a growing number of Mexican Americans complete college degrees [51], enter professional occupations [22], and build strong social and economic ties in the U.S. that reduce remittances to family in Mexico [37]. In addition, Mexican American marriage rates and marital stability are high, age at first marriage and first birth have increased, and family size has declined [50, 52, 53]. Together, changes in these traits led to increased saving rates, homeownership, business startup, and investment among Mexican Americans [54–56]. Mexican Americans tend to invest heavily in real estate, often in the primary residence but increasingly in other real estate; by contrast, financial asset ownership appears to be relatively low among Mexican Americans compared to immigrants from other regions [57]. There is some indication that financial asset ownership may be increasing for Mexican Americans [18, 22], a signal that Mexican American presence in top wealth positions may grow in the future.

By contrast, Cuban Americans are a large and visible Latino group in the U.S. that has often been referred to as high-achieving [58]; yet there is considerable heterogeneity in achievement among Cubans, suggesting that the visible members of this group may not be representative of the whole. Many of those emigrating from Cuba immediately following the Cuban revolution were highly educated, high-income, land and business owners. At least partly as a result, a small number of Cuban immigrants have become well-known for their achievements in politics, among CEOs of Fortune 500 companies, and in the Forbes 400 wealthiest families [59, 60]. Yet, subsequent waves of Cuban immigrants–including those who arrived during the Mariel boatlift in 1980 –had low- to middle- socioeconomic status (SES). Most importantly, there is little reason to anticipate a particular change in their wealth position in recent decades [23]. Indeed, Cuban education, income, and business startup levels were high in the 1980s following an influx of immigrants with high SES. However, Cuban achievement has been average in recent years, particularly among the foreign-born [61]. Other Latino groups are relatively small and despite some notable exceptions–such as the three members of the Columbian Santo Domingo family in the Forbes 400 [59]–have not experienced notable changes in their social or economic status.

Current estimates indicate that 6% of top wealth holders are from ethnic groups that are not white, black or Latino [2], and changing immigration patterns suggest that many of these top wealth holders are likely to be Chinese. Of course, there is considerable heterogeneity among Asian immigrants, including those from China, but high median values on many measures of achievement suggest that this group may be well-represented among top wealth holders. There is a long history of highly-educated, professionals immigrating from Hong Kong and Taiwan with legal status; it is also clear that these immigrants have moved into top CEO positions [60], have high overall saving rates, invest heavily in financial and business assets, and accumulate notably high levels of net worth as a result [56, 62, 63]. Consistent with this, it is likely that some top wealth holders are also from Hong Kong and Taiwan. Given that immigration from Hong Kong and Taiwan has been relatively stable in recent decades and that the demographics of immigrants from these regions has not changed notably, it is likely that the representation of Hong Kong and Taiwanese immigrants at the top of the wealth distribution has also been relatively stable.

Similarly, large numbers of top wealth holders are likely to be from Mainland China. In 2009, Asian Americans surpassed Latinos as the fastest growing segment of the foreign-born population, and much of this change reflects a growing number of Mainland Chinese immigrants [17]. In addition to increasing in numbers, the education levels and professional experience of Mainland Chinese immigrants has also increased since the 1990s as the Mainland Chinese economy has developed and the Chinese educational system has become more consistent with western educational standards. Mainland Chinese immigrants are also more likely to receive employment visas and to arrive to well-established communities of co-ethnics in the U.S. that provide an arrival context that facilitates integration and mobility. Similar to immigrants from Hong Kong and Taiwan, those from Mainland China appear to have high saving rates, often weighted more heavily toward real estate and business assets. However, financial asset ownership is increasing for Mainlanders, suggesting their presence in top wealth positions is likely to be high now and to grow in the future [56, 62, 63].

There are also likely to be Indian immigrants among top wealth holding households. Approximately 8% of immigrants to the U.S. are Indian, and immigration from India has increased in recent decades as employment visas have become more accessible to this group [64]. Indian immigrants now receive more employment visas than any other nationality, a pattern which can affect both the income and wealth of those entering the U.S. and the subsequent salaries and saving of those who obtain employment visas [17, 65]. There is considerable heterogeneity among Indian immigrants, but Indians tend to have incomes, education levels, and occupational experiences that are equal to or higher than non-Hispanic white natives [17, 41]. For example, 75% of adult Indian immigrants have at least a bachelor’s degree and only 2.3% have less than 12 years of education [56, 65]. In addition, 70% of Indian immigrants speak English comfortably compared to 49% of all other immigrants [65]. Consistent with these patterns, Indian immigrants occupy many top income positions in the U.S.: Indian males are notably overrepresented in high-salary positions in information technology, management, business, and finance; and Indian females are highly-represented in top positions in the management and finance [65]. Saving rates, investment in financial assets, homeownership, and business start-up are also notably high for Indian immigrants [62] providing further evidence that Indians may accumulate assets as well.

Finally, Korean and Filipino immigrants are likely to be well-represented among top wealth holders. The size of each of these immigrant groups has increased in recent years, and each now accounts for between 10% and 20% of the U.S. Asian population. Again, like all Asian immigrants to the U.S., there is considerable diversity within the Korean and Filipino communities on all measures of achievement; however, median achievement tends to be high compared to the overall U.S. population as a result of selection of immigrants on education and professional skills, suggesting that members of these groups may move into top wealth positions following immigration [17]. Korean immigrants have median incomes that are on par with the U.S. median (about $50,000), but 35% of Koreans have bachelor’s degrees and 18% have advanced degrees compared to 18% and 10% of all Americans. Median income for Filipino immigrants is $75,000 and 39% have bachelor’s degrees, and Filipinos are also more likely than other Asian immigrants to speak English comfortably. Of all Asian immigrants, 53% report being comfortable speaking English well, whereas 69% of Filipinos report comfort with English.

## Materials and methods

### Procedure

To study the national origin of top wealth holders, it is necessary to have a representative sample of high-wealth households with data on each respondent’s country of birth. Since large portions of net worth are owned by relatively few households, ordinary random samples of households tend to underrepresent top households. Collecting data on a sample of high-wealth households requires considerable resources because affluent households are often unwilling to reveal details about their incomes and assets to survey researchers [66]; thus, representative samples of wealthy households are rare. In addition, national origin data (i.e., a survey question indicating the country in which the respondent was born) is necessary to identify immigrants, but surveys that collect this data rarely accommodate their sampling techniques to accurately represent high-wealth households.

Because no single dataset contains both top wealth holders and data on country of birth, we use a synthetic data strategy to impute country of birth information from one dataset into a separate dataset that has a large, representative sample of high wealth households. This methodology rests on a form of multiple imputation that allows researchers to simultaneously draw on the unique strengths of two datasets [67]. Multiple imputation is more commonly used to deal with missing data in surveys and to mask respondent identities to protect confidentiality [68], but it can also be used to synthetically merge distinct datasets that have complementary strengths. This is accomplished by appending the two datasets, treating the unique variable as missing in the receiver dataset, and imputing the unique variable into the receiver set based on the two datasets’ shared variables. In this analysis, the term “partially-synthetic” refers to the fact that national origin was missing from the dataset of analysis, whereas all other variables are observed; this is consistent with census bureau terminology [69].

Multiple imputation rests on the strong assumption that missing data is missing at random (MAR); that is, patterns of missing data should be completely explained by patterns in the complete information [68]. This is usually impossible to verify statistically, and in many cases (especially when multiple imputation is used to fill missing data within a single survey), the MAR assumption is not necessarily justified. Instead, missing responses are often caused by the nature of the content within a response, which cannot be explained by observed patterns in the data. For example, high-income respondents may be reluctant to report total income or income components, but these respondents are sometimes identical to upper middle-income respondents on all other traits. This type of pattern of missing data is referred to as missing not at random (MNAR) and is highly problematic for multiple imputation. A key advantage of imputing data across datasets is that missingness is entirely due to unasked questions rather than any pattern in the responses themselves. If a question is unasked in one survey, this pattern of random missingness is referred to as missing completely at random (MCAR). MCAR is considered an ideal condition for imputing data because it prevents imputation bias from resulting from the exclusion of particular variables [67]. Due to the reliability of imputation models with MCAR data, multiple imputation is frequently used to fill MCAR data such as in split ballot surveys, anonymized data, and merged surveys, [69–71]; and estimates from this form of synthesis can be consistent with the original datasets [69].

In our study, the missing generation process is unrelated to any unobservable parameter of interest; instead, missing data was due to questions that were unasked. Moreover, the decision not to gather information on national origin in the high wealth sample was unrelated to any distinct trends among the nationality of those respondents; rather, the decision was made for confidentiality purposes. In fact, the survey containing our high wealth sample declined to even release data on race for respondents in small minority groups as it would make such respondents too easy to identify. However, the composition of our high-wealth sample is slightly different from the composition of our sample containing data on national origin, and these differences (socio-economic status) are related to the missing data (national origin). Therefore, we believe that it is reasonable to assume that the pattern of missing data for national origin is MAR, which satisfies the assumptions of multiple imputation. Multiple imputation across surveys is fairly robust to slight differences in sample composition [67], but as described in the data section below, we conducted many analyses to ensure that the MAR assumption is not violated.

All imputations in our work were conducted with the Fully Conditional Specification (FCS) method. FCS has various names including the Sequential Regression Multivariate Imputation (SRMI) method and the Multivariate Imputation by Chained Equations (MICE) method. The FCS method differs from its most common alternative, the Multivariate Normal (MVN) method, in that it does not assume that variables are jointly distributed [72]. Instead, FCS imputes each variable sequentially with a regression function for continuous variables and a discriminant function for categorical variables. Allowing for discriminant function analysis is one of the key advantages of the FCS imputation method: discriminant function analysis estimates the probability of group membership for categorical variables and classifies observations into a particular membership based on least squares estimation. Although there are ways to impute categorical variables with MVN [73], research suggests that FCS is preferable for imputing a mix of continuous and categorical variables [72].

Coding and related procedures are described in more detail below, however, the variables included and the number of imputations used in the model were determined by a combination of theory and induction. To assess and refine the efficiency of our imputation model, we conducted extensive internal and external validation and sensitivity analyses. Final estimates were based on 100 imputations, and sensitivity tests indicated that additional imputations did not improve model efficiency (consistency). Because we only impute a single missing variable across the two datasets (national origin), there was no pattern of missingness in the data (a monotonic missing pattern), resulting in highly efficient imputations. As in previous research [74], the model’s reliability was tested through a series of simulations, where responses to national origin in the donor set were repeatedly masked, imputed, and compared to the real data. These tests revealed little systematic bias in the final model; aggregate estimates of national origin were relatively consistent with those in the real data, and the model was fairly resistant to increases in missing data. Given the model’s efficiency and use of FCS, model convergence (assessed with time series graphs across iterations) was fast. Demographics constructed from the synthetic data were also compared with known population demographics from the Survey of Consumer Finances, Survey of Income and Program Participation, and other publicly-available data, and were found to be relatively consistent.

The donor data contained few missing values, but these were imputed to ensure that all values were present before merging the datasets. Because the receiver dataset contained no missing values, no imputation was required within the receiver dataset. To ensure the reliability of wealth-related analyses, final analyses are only based on the receiver dataset which contained detail household information on wealth. In other words, estimates of the national origin of top wealth holders are based entirely on the synthetic implicates in the receiver dataset. In addition, all analyses incorporate the receiver dataset’s weights for post-stratification. To maximize the robustness of our estimates and leverage the multiple imputation framework, all statistics were derived from a repeated inference strategy. Results were obtained by estimating means across each replicate individually, pooling the results, and adjusting the estimates based on the variance between each replicate [68].

### Data

Data come from two sources:

1. *The Survey of Consumer Finances (SCF)* is collected triennially by the Board of Governors of the Federal Reserve and publicly-available at https://www.federalreserve.gov/econresdata/scf/scfindex.htm. The SCF is widely-regarded as the most accurate data on top income and net worth households because it includes both (a) a multistage national area probability sample and (b) a second sample of high-income households based on a blend of area-probability sampling and stratified probability sampling from Survey of Income tax returns. The multistage national area probability sample ensures representation of a cross-section of households, their assets and debts, and their demographic traits. The sample of high-income households is identified with Internal Revenue Service data [75, 76] and is calibrated against other known data to ensure accurate representation of affluent households [7, 66, 71]. Although the high-income respondents are not specifically chosen to be high-net worth, the resulting sample includes households at the top of both the income distribution and the net worth distribution [76]. The sample design ensures that the unique asset and debt ownership of top households (e.g., ownership of corporate stock, bonds, and alternative investments) and their demographic traits are represented [77].
  The SCF also contains detailed, comprehensive data on earnings, assets, debts, related financial behaviors, work behaviors, household composition, and demographic information including race (white, black, Latino, other), marital status, age, and education [78]. However, an important drawback of the SCF is that national origin (i.e., country of birth) is not included. The goal of this paper is to use a synthetic data strategy to impute national origin into the SCF from a separate survey (described below). Analysis is based on a single, pooled version of all data from 1995–2004 to assess changes over time.
2. *The Survey of Income and Program Participation (SIPP)* is a multipanel, nationally-representative survey of U.S. households collected by the U.S. Census Bureau and publicly-available at http://www.census.gov/sipp/. Panels of 14,000 to 36,700 households have been surveyed every two to four years from 1983 through 2013. Unlike the SCF, the SIPP does not include a separate sample of high-wealth households; therefore, it is not sufficient to use only the SIPP to study affluent households. The advantage of the SIPP is that it contains respondent’s national origin plus excellent data on household wealth for each panel to allow us to match SCF households based on demographics and asset holding.
  Analysis is based on a single, pooled version of all cross-sectional SIPP data from the first wave of each survey conducted from 1996–2004 to assess changes over time. SIPP data are available through 2013, but national origin questions were removed from the public data starting in 2008. Using more current data would be ideal, and we have worked with a Census Research Data Center (CDRC) to gain permission to access the more recent SIPP data. CDRC regulations prevent us from running the exact models reported here on the non-public data, but other work shows similar patterns to those reported below [79].

The SCF and SIPP are very similar post-stratification, but the socioeconomic composition of their samples are distinct, which is problematic for our procedure. Due to our interest in high-wealth households, we attempted to reduce the difference in the SCF and SIPP’s sample designs by restricting both samples to households with a net worth of at least $100,000. This threshold is relatively low (i.e., the top one percent of wealth holders owns net worth valued in the millions of dollars), but it ensures that our estimates are not weighted by information from low-wealth households. Table 1 illustrates that the resulting samples, when unweighted, are similar on most other demographic traits. Consistent with its high wealth sample, the SCF has a slightly younger, more educated sample and a higher rate of marriage than the SIPP. The SCF has more male household heads than the SIPP, but this also reflects a difference between the SCF and SIPP’s sample design; when weighted, the rates of male household heads are nearly identical between the datasets. Employment patterns are central to wealth ownership, and as the Table 1 illustrates, employment rates between the two samples are similar. There are differences in respondent racial identification between the SCF and the SIPP; however, the difference is minimal and sensitivity analyses indicate it does not affect our estimates.

**Table 1:** Comparison of SIPP and SCF Data: Household Head Demograhic Traits (%) by Year.

Year	1995/1996			2001			2004		
Survey	SIPP	SCF	Difference	SIPP	SCF	Difference	SIPP	SCF	Difference
**Age of Head**									
25–39	13.8	14.5	-0.8	13.2	12.6	0.6	14.2	11.2	3.0
40–49	22.3	23.4	-1.1	22.9	26.9	-4.1	22.0	22.9	-0.9
50–59	20.9	23.1	-2.1	22.7	25.4	-2.7	24.0	28.9	-4.9
60–69	17.4	20.0	-2.6	16.6	16.4	0.2	17.2	19.3	-2.1
70 & over	25.0	18.5	6.6	24.1	18.1	6.0	22.1	17.3	4.8
**Education of Head**									
Less Than HS	12.3	8.2	4.1	9.1	7.2	1.9	5.3	5.7	-0.4
HS Graduate	28.4	21.3	7.1	25.9	17.5	8.5	25.3	17.9	7.3
Some College	27.0	19.1	7.9	28.5	17.5	11.0	34.4	16.0	18.4
Bachelors	18.9	24.1	-5.2	21.7	27.8	-6.1	21.3	28.1	-6.8
Advanced Degree	13.3	27.2	-13.9	14.7	30.0	-15.3	13.8	32.3	-18.5
**Gender of Head**									
Female	22.6	13.1	9.5	21.0	12.3	8.7	22.0	11.8	10.2
**Race of Head**									
White	88.8	90.8	-2.0	86.6	91.2	-4.7	84.6	88.3	-3.8
Black	4.6	3.1	1.5	5.4	3.6	1.8	6.2	4.2	2.1
Latino	3.7	2.0	1.7	4.3	2.7	1.6	4.2	3.8	0.4
Other	2.8	4.1	-1.3	3.7	2.5	1.3	5.0	3.7	1.3
**Marital Status**									
Married	66.8	78.3	-11.5	66.0	78.8	-12.8	65.8	78.6	-12.8
**Employment Status**									
Unemployed	5.4	3.5	1.9	5.0	3.1	1.8	5.1	3.9	1.2

Note: Estimates based on unweighted SCF and unweighted SIPP (years 1995–2004). Cells indiciate the percent of household headsin each dataset with the specified trait.

Since a multiple imputation model rests on the multivariate distribution of its variables, we also compared the bivariate distribution among each variable in the SCF and SIPP. The correlation of each variable with all others across each dataset were fairly consistent; the average absolute difference in bivariate correlations for each variable across the datasets was .05. A few bivariate correlations differed more significantly than others (mainly among binomial variables that had low probabilities of occurrence), but only 3% of all bivariate correlations across the two datasets differed by more than .20.

### Variables used in imputation

The focal variable in the imputation model was national origin. Although immigrants in the SIPP came from over 100 different countries, the models’ discriminant function analysis requires that each classification of this variable have a sample size exceeding the number of predictor variables, preferably by a large margin [80]. Therefore, respondents were only classified into the national origins of this paper’s interest: American, European, Canadian, Mexican, Cuban, Hong Kong Chinese, Taiwanese, Mainland Chinese, Asian Indian, Korean, and Filipino. Ideally the results would include separate estimates for Hong Kong and Taiwanese immigrants, but the Taiwanese sample in the SIPP is relatively small, and SIPP respondents from these two groups were similar on most variables used in our analyses. We ultimately decided to merge the Hong Kong and Taiwanese groups, consistent with standards in the immigration literature [56]. Unfortunately, neither the SCF nor the SIPP include generation status, making it impossible to distinguish immigrants by generation. All other national origin indicators were merged into a single “other national origin” category. This was necessary but violates a key assumption of discriminant function analysis: homogeneity of variances/covariances [81]. In other words, the “other national origin” category contained subpopulations that had distinct correlation matrices among the model’s predictors. The heteroscedasticity of this category prevented the model from ever imputing respondents into it. Instead, most observations from the other national origin category were imputed as American born.

Validation tests revealed that the model had a slight bias to impute immigrants as American-born among all national origin groups. In total, about one-third of the immigrant sample was imputed as American born in our validity tests. As a result of this systematic bias, our estimates of immigrants in the one percent are somewhat conservative. However, two national origin groups required additional measures to ensure that the model’s estimates were accurate. The initial model underestimated the number of Asian Indian and Cuban households in masked portions of the SIPP. Outlier tests determined that the predictive power of the model was substantially hampered by a small number (approximately 10%) of Asian Indian households that reported their race as white, and Cuban households that reported their race as non-Latino. These patterns likely reflect unique ethnoracial patterns that have been documented for these groups [58, 82]. A random decision forest determined that race was by far the model’s most important variable for classifying households into national origins. Since race was a weak predictor for Asian Indian and Cuban households, the model used an overly-conservative criteria for imputing households into those national origins. To address this problem, we removed white Asian Indian households and non-Latino Cuban households from our sample. Even though these households could represent a realistic portion of immigrants from those national origins, they introduced an unacceptable bias in the imputation model. Removing these households introduced a new bias into the model such that no white households were imputed as Asian Indian and no non-Latino households were imputed as Cuban. However, the resulting model was several times more accurate and consistent at imputing Asian Indian and Cuban households than it was previously.

Our independent variables were chosen based on theory and induction. We first identified all demographic and financial variables that are included in both the SCF and the SIPP and that are correlated with national origin. This resulted in a list with more than 100 variables; however, over half of these variables were not sufficiently similar between the SCF and SIPP to enable comparison. A few variables that were correlated with national origin were culled from the final list because they were deemed spurious (i.e. they were not supported by previous research). Importantly, although both datasets include wealth, we omitted most wealth measures at this stage because the SIPP does not contain a representative sample of high wealth households [83]. Therefore, including measures that were highly correlated with wealth in the imputations would introduce systematic bias for high-wealth households. Other wealth measures were kept in the model because they were correlated with national origin but were not highly correlated with total net worth or significantly influenced by outliers. Each dataset includes sample weights, but these were not incorporated into the model because all of the variables used to generate the weights were already in the model [67].

Based on research about the human capital and racial perception of people from differing national origins, the model’s independent variables included categorical variables for education (less than high school education, high school graduate, some college, bachelor’s degree, and advanced degree) and race (white, black, Latino, and other) [84]. There is strong evidence that our immigrant groups have distinct family structures, therefore we incorporated variables for marital status (never married, married, widowed, divorced, separated, and neither separated nor cohabitant), respondent and spouse work status (full time, part time, and other), age (years), household size (capped at eight), number of children (capped at six), whether the household was headed by a female, and whether either household head’s parents lived in the household [85].

Previous research suggests a few important distinctions in the financial and consumption habits of immigrant households [56], therefore we included count variables for the number mortgages and vehicles that respondents owned, and dummy variables for whether respondents owned at least one home, interest bank account, savings account (IRA, 401k, or Keogh), or vehicle (detailed by type). Several logged continuous variables for money in assets (savings plans, vehicles, and investment real estate) and debt (secured, consumer, vehicle) were also included in the model. In addition, to account for any time trends reflected in the SIPP’s national origin numbers, we included a continuous variable for the year in which each observation was gathered.

All demographic and financial variables used to impute national origin in the SCF and SIPP were recoded to be equivalent. Because multiple imputation techniques with discriminant function analysis rest on an assumption that variables are normally distributed, we followed standard practice and logged all continuous variables that had non-normal distributions, capped continuous variables that had long tails, and merged categories within categorical variables that were rare (less than 5% occurrence). Sensitivity analyses indicated that the results do not reflect the presence of outliers.

## Results

There is little doubt that top wealth holders in the United States are predominantly white, but consistent with our expectations, our results suggest that many of these elite households are likely to be members of the growing population of foreign-born Americans. Table 2 provides estimates of the national origin of top wealth-holding households. We include estimates for those in the top one percent and the top five percent to illustrate the difference between the composition of high wealth holders in the U.S. and the most elite. Since the one percent controls such large portions of wealth and are often the focus in prior literature, we focus on the one percent in all subsequent tables. Our dollar cutoffs for membership in the one percent and five percent are consistent with other research based on the SCF [2, 7]: it took $7.8 million and $1.8 million in net worth respectively to be in the top one and top five percent of households in 2004. All dollar values are in 2013 currency. Per our discussion of the literature, we show results for the native and foreign-born ethnic groups that are most likely to be represented among top wealth holders, although our data could allow for additional detail regarding national origin. To increase statistical power, we present findings that are pooled over time (1995–2004). This decision is in line with our preliminary exploration and prior research, which suggest that there had been few changes in the ethnic composition of the elite over that decade.

**Table 2:** The National Origin of Top Wealth Holders (%).

	Top 1%		Top 5%	
	Mean	S.E.	Mean	S.E.
**United States**				
Native White	91.53	0.89	90.40	0.69
Native Black	0.92	0.19	1.07	0.19
Native Latino	0.79	0.33	1.27	0.24
Native Asian	1.21	0.35	1.18	0.29
**Caucasian**				
European	2.76	0.71	2.88	0.53
Canadian	0.61	0.35	0.63	0.26
**Latin America**				
Mexican	0.43	0.20	0.27	0.12
Cuban	0.05	0.09	0.10	0.12
**Asia**				
HK/Taiwan	0.27	0.21	0.41	0.21
Mainland Chinese	0.65	0.26	0.53	0.21
Asian Indian	0.51	0.24	0.73	0.23
Korean	0.12	0.14	0.27	0.17
Filipino	0.15	0.17	0.27	0.17

Note: Estimates based on weighted SCF synthetic dataset (replicates = 500, years 1995–2004). Cells indicate the percent of each wealth group from the specified national origin.

It is important to interpret all of our results as estimates rather than sample statistics given that they reflect findings from our synthetic dataset; however, estimates from merged data have been shown to be robust, and our own merged data effectively reproduce estimates of other household traits and aggregate patterns in each component dataset [69]. Consistent with most research on top wealth holders [2], Table 2 shows that the majority of affluent households are native-born whites (92%) and very few are native-born black (1%). However, as we anticipated, Table 2 shows that at least some of the top wealth owners (3%) who are typically classified as white and assumed to be native-born are likely to be European or Canadian immigrants [2, 7, 9]. Similarly, our results suggest that about 3% of households in the top five percent of wealth owners are probably white but of European or Canadian origin. Although 3% is a relatively small *percentage* of households, this includes a substantial *number* of households with considerable resources and corresponding influence. In the years we used to generate these findings, there were slightly more than 1 million households in the top one percent of wealth owners in the U.S. (authors’ calculations). Remittances from the United States to Europe and Canada are relatively high, and immigrants from these regions–particularly high-wealth households–also tend to invest their own resources in assets such as real estate and businesses in the home country at relatively high rates. Thus even small percentage of top households with strong ties to other countries implies that wealth ownership is more global than previous estimates suggest.

As we anticipated, changing immigration dynamics are leading to change in the composition of those at the top of the wealth distribution. For example, we know from previous research that approximately 1.2% of top wealth holders are Latinos, and Table 2 shows that native-born Latinos likely account for the largest portion of Latinos in the one percent (.79%) and an even larger portion of the top five percent of wealth holders (1.27%). But perhaps more importantly, our results show that there are likely to be Mexican and Cuban immigrants who rank among the highest wealth holders in the U.S. We find that .43% of the top one percent and .27% of the top five percent of wealth holders are likely Mexican immigrants, a group that is selected to have traits that lead to wealth ownership. Again, these are fairly small percentages, but they represent substantial numbers of households who have accumulated extremely high wealth. This finding also speaks directly to the immigration literature, in which a consistent debate surrounds the prospects for Mexican immigrants doing well financially in the U.S. In contrast to a theoretical contingent which argues that Mexican immigrants are mostly downwardly mobile, our findings imply that there are large numbers of Mexican immigrants among the very wealthiest households in the U.S. Given that wealth can be passed to future generations and that Mexican immigrant business owners often pass businesses to their children [22], the presence of Mexican-origin households in the elite directly contradicts the notion that Mexican immigrants are destined to the underclass.

Cuban immigrants are also represented among top wealth holders, although there are fewer Cuban than Mexican households in the one percent, a pattern which, at first glance, may seem surprising. Table 2 shows that only .05% of the top one percent and .10% of the top five percent of households are Cuban immigrants. However, this finding is consistent with recent immigration dynamics and with literature on household financial well-being in the U.S. The small number of visible Cubans who attract attention in politics and business have influenced public perception of Cuban immigrants as a high-achieving minority group. Yet there is considerable within-group heterogeneity among Cuban Americans; many recent Cuban immigrants have been middle and working class. In addition, our estimates reflect first generation immigrants rather than the high-SES post-revolution immigrants or their potentially high-achieving children. Therefore, it is no surprise that our estimates include only small numbers of high-wealth Cubans.

Asian immigrants are well-represented in both the top one percent and the top five percent, consistent with our expectations. There is a long history of Chinese immigration to the U.S., and a recent influx has attracted attention to the particularly high-achieving segment of the immigration population [64]. Hong Kong and Taiwanese immigrants are the smaller and more heterogeneous segment of this large group, but our estimates show that these households are, nonetheless, well-represented among top wealth holders. Table 2 shows that 27% of the top one percent and .41% of the top five percent are Hong Kong/Taiwanese immigrants. Mainland Chinese immigrants are highly-selected for educational and professional achievement and are the largest group of Asian immigrants, therefore we anticipated and found that even larger percentages of top U.S. wealth holders are Mainlanders: .65% of the top one percent and .53% of the top five percent are Mainland Chinese. Similarly, Asian Indian immigrants–whose educational and professional selection are similar to that of Mainland Chinese–comprise .51% of the top one percent and .73% of the top five percent of wealth owners. For both Mainland Chinese and Asian Indian immigrants, these numbers may seem small given the well-publicized and thoroughly-documented success of many members of these groups, but again, these small percentages represent sizable numbers of households. Moreover, the large influxes of immigrants from these countries is relatively recent, and many immigrants are young and either still in school or recently graduated from universities and graduate programs. Both groups are likely to be even better represented in top wealth positions in the future. Finally, as we anticipated, there are Korean and Filipino immigrants in top U.S. wealth positions as well, but their numbers are smaller than for other Asian subgroups. Our estimates suggest that .12% and .15% of the top one percent are Korean and Filipino respectively, while .27% of each top group are from these groups.

The allocation of assets across financial instruments can affect the wealth accumulation and corresponding wealth status (i.e., membership in top groups) for both current and future generations. The typical American household—if they save at all—tends to accumulate assets in the primary residence. Investment in financial assets is less common for the average household. In contrast, high-wealth households often own real estate apart from the primary residency and typically own large amounts of various financial instruments. Given that the returns associated with financial asset ownership can be much sizable, financial asset ownership is usually associated with much higher overall asset accumulation. To explore whether there are national origin differences in asset allocation that might imply different accumulation trajectories, we estimated the national origin of top non-financial and top financial asset owners separately. Table 3 illustrates the percent of top non-financial and financial owners (top one percent) who are members of the specified national origin groups; non-financial assets include all tangible assets (e.g., real estate, businesses), whereas financial assets include all monetary assets (e.g., cash accounts, stocks, bonds, and retirement accounts). Our findings indicate that native-born white households dominate top financial asset positions even more than they do top non-financial asset positions; however, immigrants are present in both top financial groups.

**Table 3:** The National Origin of Top Non-Financial and Financial Asset Owners (for those in the One Percent).

	Non-Financial		Financial Assets	
	Mean	S.E.	Mean	S.E.
**United States**				
Native White	88.70	0.99	93.34	1.07
Native Black	1.30	0.25	0.21	0.11
Native Latino	0.86	0.28	0.52	0.22
Native Asian	1.63	0.48	0.93	0.37
**Caucasian**				
European	2.78	0.73	2.89	0.80
Canadian	0.57	0.35	0.67	0.38
**Latin America**				
Mexican	0.72	0.25	0.09	0.13
Cuban	0.06	0.10	0.04	0.09
**Asia**				
HK/Taiwan	0.53	0.34	0.25	0.22
Mainland Chinese	1.28	0.47	0.25	0.21
Asian Indian	0.89	0.41	0.56	0.24
Korean	0.31	0.23	0.12	0.16
Filipino	0.36	0.28	0.12	0.17

Note: Estimates based on weighted SCF synthetic dataset (replicates = 500, years 1995–2004). Cells indicate the percent of each financial group from the specified national origin, for those in the one percent.

For most of the groups we highlight, Table 3 shows that representation in top non-financial and top financial asset groups is comparable to their representation in top net worth positions; however, two important exceptions are worth noting. First, there are more Mexican immigrants in top non-financial asset positions than in top net worth positions, consistent with our expectations about real estate and business ownership among Mexican immigrants. On the other hand, Mexican immigrants are comparatively underrepresented among top financial asset owners. Mexican geographic proximity to the U.S. eases immigration and reduces selection making Mexican immigrants among the most disadvantaged immigrant groups. Yet like nearly all immigrants, Mexican immigrants tend to be more highly educated, more occupationally motivated, and to have more entrepreneurial inclinations than others from the home country. Consistent with our expectations and previous research [18, 22], our findings suggest that business and financial asset ownership are the vehicles through which some Mexican immigrants are incorporating. The relatively low level of financial asset ownership likely reflects delayed incorporation into the financial system; clearly our results show that some Mexican Americans are overcoming this, however, a pattern which suggests that financial connectivity is on the rise. Second, Mainland Chinese immigrants also account for a larger portion of top non-financial asset owners than of top financial asset owners. For Mainlanders, this pattern reflects the age of immigrants who are, in many cases, still finishing school and are still likely to begin saving and investing in financial assets.

Our final tables take a slightly different approach to understanding how national origin is associated with asset allocation. Tables 4 and 5 identify asset classes and show the percent of total assets invested in each class for those in the top one percent of net worth holders. That is, we start with the households identified as the top one percent in Table 2, and we look at how they invest their assets. These estimates show basic differences by national origin in investment patterns and strategies, and they can also be suggestive of future trends. For instance, a tendency to weight investments more heavily toward financial assets–particularly stocks–can lead to more rapid wealth accumulation that will secure high-wealth status for current and future generations. Of course, real estate investments can also be lucrative and can lead to security through their use-value and by ensuring a portfolio is diversified. Table 4 shows the percent of assets invested in total real estate and total financial assets and illustrates that there are meaningful differences across national origin groups in investment patterns. Consistent with the patterns shown in Table 3, Mexican and Mainland Chinese invest relatively high percentages of their assets in real estate; by contrast, Cuban immigrants invest relatively small portions of their assets in real estate. For the other groups on which we focus, real estate investments account for approximately 20% of total assets, similar to white natives. Table 4 also shows that white and Asian natives, European and Canadian immigrants, and Asian Indian immigrants all invest approximately 45% of their total assets in financial assets. Consistent with the patterns shown in Table 3, Mexican and Mainland Chinese invest relatively low percentages of their assets in financial assets.

**Table 4:** Asset Allocation of Top Wealth Owners: Proportion of Assets in Real Estate and Financial Assets.

	Real estate		Financial Assets	
	Mean	S.E.	Mean	S.E.
**United States**				
Native White	0.21	0.01	0.44	0.01
Native Black	0.17	0.05	0.26	0.07
Native Latino	0.20	0.08	0.34	0.13
Native Asian	0.24	0.05	0.43	0.09
**Caucasian**				
European	0.21	0.06	0.43	0.09
Canadian	0.20	0.11	0.46	0.18
**Latin America**				
Mexican	0.22	0.08	0.21	0.12
Cuban	0.14	0.20	0.38	0.29
**Asia**				
HK/Taiwan	0.22	0.13	0.38	0.18
Mainland Chinese	0.65	0.17	0.17	0.11
Asian Indian	0.20	0.08	0.46	0.11
Korean	0.22	0.20	0.35	0.27
Filipino	0.27	0.23	0.28	0.22

Note. Estimates based on weighted SCF synthetic dataset (replicates = 500, years 1995–2004). Cells indicate the proportion of total assets allocated to each asset class from the specified national origin.

**Table 5:** Asset Allocation of Top Wealth Owners: Proportion of Total Net Worth in Stocks, Bonds, and Retirement Accounts.

	Stocks		Bonds		Retirement Accounts	
	Mean	S.E.	Mean	S.E.	Mean	S.E.
**United States**						
Native White	0.18	0.01	0.04	0.00	0.05	0.00
Native Black	0.02	0.02	0.00	0.01	0.01	0.01
Native Latino	0.07	0.05	0.00	0.00	0.02	0.01
Native Asian	0.07	0.04	0.02	0.03	0.16	0.06
**Caucasian**						
European	0.17	0.06	0.04	0.03	0.05	0.03
Canadian	0.18	0.13	0.04	0.05	0.05	0.07
**Latin America**						
Mexican	0.02	0.04	0.00	0.01	0.00	0.02
Cuban	0.18	0.20	0.03	0.12	0.03	0.04
**Asia**						
HK/Taiwan	0.10	0.11	0.03	0.05	0.04	0.06
Mainland Chinese	0.06	0.06	0.01	0.02	0.01	0.02
Asian Indian	0.13	0.06	0.01	0.02	0.05	0.03
Korean	0.16	0.25	0.05	0.12	0.03	0.07
Filipino	0.08	0.16	0.07	0.14	0.03	0.08

Note: Estimates based on weighted SCF synthetic dataset (replicates = 500, years 1995–2004). Cells indicate the proportion of total net worth invested into each asset from the specified national origin. Stocks includes stocks and mutual funds. Retirement accounts include 401K Accounts, Individual Retirement Accounts, Keogh Accounts and similar plans.

Table 5 provides additional information about differences in asset allocation by identifying the three largest financial asset classes and illustrative national origin differences in investment in these classes. The Table shows the proportion of total net worth invested in each asset class by national origin. Stocks and stock mutual funds are the dominant financial asset class for white natives and European and Canadian immigrants, two groups that we have seen tend to invest similarly and to dominate top wealth positions. However, Table 5 also shows that Cuban and Korean immigrants are unique in their tendency to hold stocks and stock mutual funds. Additional analyses (not shown) indicate that for Cubans, this group contains disproportionate numbers of older immigrants who were successful in business; while for Korean immigrants, the heavy stock investors are more likely to be young professionals who immigrated with assets and have not invested in more permanent forms of wealth such as real estate. Table 5 also illustrates investments in bonds and retirement accounts (including 401K and similar company-sponsored retirement accounts, Individual Retirement Accounts, and Keogh Accounts). Native Asians have been shown to have a high propensity to invest in retirement accounts [62], and our results are consistent with such findings. Finally, Mexican immigrants are noteworthy again for their low investment in bonds and retirement accounts; again, this pattern is consistent with selection of immigrants from Mexico and with previous literature documenting a propensity to invest in business in real estate for Mexican immigrants.

## Discussion

Although trends in wealth ownership and inequality are now taken-for-granted, we are only beginning to understand who has access to the positions at the top of the wealth distribution where a considerable majority of total assets are held. One of the most striking gaps in this literature has been the absence of evidence regarding the extent to which immigrants occupy these elite positions. Owning large amounts of wealth not only provide immigrant groups many social, political, and economic advantages, but it can signal long-term changes in their economic well-being. In addition, ownership of certain assets–such as the home or a business–is an important signal of immigrants’ mobility and cultural assimilation [22–24]. Our work suggests that immigrants are well-represented in top positions and, perhaps more importantly, are poised to expand their presence among the most affluent and powerful households in the U.S. These results contribute to understanding wealth ownership and inequality by providing ethnic and nationality details about the most influential households in the U.S. They also suggest that the distribution and wealth and related asset accumulation processes are much more global than previous inequality research suggests.

Although there is little question that the majority of top wealth owners in the United States are white and native-born, immigration to the U.S. is substantial, and migrants tend to be selected for attributes that are positively correlated with saving and asset accumulation. It is probable that a large numbers of top wealth holders may be immigrants, but data constraints have prevented researchers from estimating the representation of the elite who are immigrants. In this paper, we provided the first detailed estimates of national origin of top wealth holders in the U.S. Because no single survey dataset includes information on both top wealth holders and information about country of origin, we used two datasets to estimate the national origin of top wealth holders: the Survey of Consumer Finances (SCF) and the Survey of Income and Program Participation (SIPP). With these datasets, we used an innovative multiple imputation strategy to impute country of birth from the SIPP into the SCF.

Consistent with historic patterns of immigration, our results suggest that large numbers of top wealth holders who are typically thought to be white natives may well be immigrants from European countries and Canada. Specifically, we found that approximately 3% of the top wealth owners who are classified as white and who are typically assumed to be native-born are more likely to be European or Canadian immigrants. Although 3% is a relatively small *portion* of households, this is a fairly large *number* of households and individuals who have considerable resources and corresponding influence. This finding is consistent with what we know about immigrants from these regions. Not only have these regions been the traditional source of immigration to the U.S., but European and Canadian immigrants have also tended to have high levels of education and work experience that enable them to enter professional occupations, earn high salaries, and accumulate significant assets following migration (CITE). European and Canadian immigrants to the U.S. are also likely to have accumulated some savings before migrating and to use those assets as a foundation for accumulating additional assets (e.g., real estate, business, or financial assets) in the U.S (CITE).

We also proposed whether changing immigration dynamics have altered the composition of the elite, and our results were consistent with our expectations. Since the 1970s, immigration to the U.S. is no longer dominated by European and Canadian migrants. Rather, today’s immigrants are more likely to arrive from Latin America and Asia, reflecting changes to U.S. immigration law. Because these households are being selected on traits that are associated with wealth accumulation, it follows that they will be well-represented among top wealth households. In particular, we found that notable numbers of top wealth holders are likely to be Mexican immigrants. In fact, our results suggest that a somewhat larger portion of the one percent and five percent are Mexican rather than Cuban immigrants. This may surprise some readers, but these findings are supported by empirical trends. Following the Cuban revolution, many Cuban immigrants came to the U.S. with high levels of human capital and resources, but the achievement of more recent Cuban immigrants has been average. On the other hand, while Mexican immigrants tend to be disadvantaged when they arrive in the U.S., even compared to other immigrants, recent evidence finds that Mexican immigrants are increasingly likely to graduate from college [51], take professional jobs [22], and reduce remittances over time [37]. Wealth tends to increase in married couples and decline with family size, and recent evidence shows that Mexican American marriage rates and marital stability are high, age at first marriage and first birth have increased, and family size has declined [50, 52, 53].

Our findings regarding Mexican immigrants are inconsistent with research that argues that Mexican immigrants are *the* textbook example of a downwardly mobile immigrant group as the result of low human capital and negative public reception, but our findings support a growing body of evidence that suggests that Mexican immigrants are often upwardly mobility [18, 22, 86]. Mexican geographic proximity to the U.S. eases immigration and reduces selection, making Mexican immigrants among the most disadvantaged immigrant groups, yet like nearly all immigrants, Mexican immigrants tend to be more highly educated, more occupationally motivated, and to have more entrepreneurial inclinations than others from their home country. This is somewhat reflected by our findings regarding business and financial asset ownership among immigrant groups. Mexican immigrants had relatively low rates of financial asset ownership, which reflects their delayed incorporation into the financial system; however, our results show that some Mexican Americans are overcoming this–a pattern which suggests that their financial connectivity is on the rise.

We also found evidence that a significant portion of top-wealth owning households who are usually classified as having an unspecified ethnicity are likely to be Asian immigrants, including immigrants from Hong Kong, Taiwan, Mainland China, India, Korea, and the Philippines. There is considerable heterogeneity among Chinese immigrants, but education levels, professional experience, and familiarity with business start-up are all relatively high for immigrants from Hong Kong, Taiwan, and Mainland China. Moreover, immigrants from these countries are likely to be documented, adding to the job opportunities and wealth accumulation potential that they encounter upon arrival. Changing patterns of immigration from Mainland China are particularly interesting for our purposes: Immigrants from Mainland China are increasing in number, and immigrant education levels and professional experience have both increased in recent years consistent with changing economic development in the home country [17]. Our findings suggest that immigrants from Mainland China are benefitting from these advantages and moving into top wealth positions. Similarly, immigrants from India tend to be documented and to have high educations and professional experience. Indian immigrants are somewhat unique in their receipt of employment visas that enable them to take highly-paid professional occupations [17, 65]. These traits lead to high saving rates that are the likely mechanisms leading to the large number of Indian immigrants we estimate among top wealth holders. In addition, we speculated that Korean and Filipino immigrants are likely to be well-represented among top wealth holders because the size of each of these immigrant groups has increased in recent years; and although there is significant diversity within the Korean and Filipino communities on all measures of achievement, median achievement tends to be high compared to the overall U.S. population as a result of selection of immigrants on education and professional skills [17]. Our results suggest, again, that these immigrants may be making inroads into top wealth positions that will create household-level advantages and class-wide mobility and stability.

Our findings contribute to the immigration literature by offering new insight into the financial well-being of some of the largest groups of immigrants in the U.S. In particular, our results contradict the common assumption in the immigration literature that Mexican immigrants are destined to remain in the underclass. They also contradict the common perception that Cuban immigrants are a somewhat homogeneous and high-achieving ethnic minority–our findings show that some Cubans achieve top wealth status, but the representation of Cubans in top positions is modest. The large number of Asian immigrants–particularly Chinese and Indian immigrants–in top positions perhaps foreshadows a growing presence of these groups in top positions. Since there are significant economic, social, and political influence that can accompany the ownership of large amounts of wealth is significant, even a small proportion of households with high wealth and interests that diverge from those of the majority elite can be meaningful. Political influence is an important example. The political interests of immigrants may be different from those of natives; if even a portion of top wealth-owning immigrants use their financial resources to support certain candidates, election results may be affected.

Our findings are suggestive of important patterns, but there are ways that future research will want to improve on this work. Notably, our data strategy was a response to the lack of data from a single source on the national origin of top wealth holders. Including information on national origin in data such as the SCF that surveys top wealth holders would allow researchers to document these patterns with more authority. Ideally, we would also have much larger samples and longitudinal information on the same households to understand trends in wealth accumulation over time. Finally, future research could usually document the degree to which high net worth households hold assets in a single country or globally. The presence of immigrants in top wealth positions in the U.S. suggests a growing globalization of asset ownership, but ideally, we would have data that allows us to study this directly. There are data sets that contain information about asset holding by immigrants in other countries (e.g., the New Immigrant Survey), but like most data sets, these do not have sufficient samples of top wealth holders to allow focus on the households that control most wealth.
